# Atrial Fibrillation and Transient Ischemic Attack Encountered in the Management of Snake Bite

**DOI:** 10.4021/cr106e

**Published:** 2010-11-20

**Authors:** Ozgur Sogut, Halil Kaya, Mehmet T Gokdemir, Mustafa B Sayhan, Nurkal Halis

**Affiliations:** a Department of Emergency Medicine, Harran University, Faculty of Medicine, Sanliurfa, Turkey; b Selimiye State Hospital, Emergency Service, Edirne, Turkey; c Department of Emergency Medicine, Research and Training Hospital, Bursa, Turkey

**Keywords:** Snake envenomation, Antivenom, Epinephrine, Atrial fibrillation, Transient ischemic attack

## Abstract

Thousands of snake bites occur in the world each year, with hundreds of patients receiving antivenom. Incidence rates for immediate hypersensitivity reactions associated with the use of antivenom range vary. This is a case report of a patient with atrial fibrillation (AF) and transient ischemic attack (TIA) induced by vigrous snake bite that was suspected to be caused by the treatment of subcutaneous epinephrine, due to immediate hypersensitivity reaction following the administration of antivenom treatment.

## Introduction

Management of envenomed snake bites is a difficult task. The envenomation mechanism in snake bites are still unsatisfactory. The definitive treatment for severe envenomation is antivenom [1, 2]. There have been numerous reports of immediate hypersensitivity reactions associated with the use of antivenom [2-4]. Incidence rates for immediate hypersensitivity reactions associated with the use of this product range from 23% to 56% [1]. Anaphylaxis to the treatment of antivenom varies according to the patient's response and nature of the insult [4].

Herein, we present a case of atrial fibrillation (AF) and transient ischemic attack (TIA) with vigrous snake bite that was suspected to be caused by the treatment of subcutaneous epinephrine, due to immediate hypersensitivity reaction following the administration of antivenom treatment.

## Case Report

A 69-year-old woman was presented to our emergency department (ED) with rapidly progressing right upper extremity pain, edema, and ecchymosis after snakebite. She had neither been bitten by a snake nor received antivenom in the past. The patient was a non-smoker, with a history of non-insulin dependent diabetes mellitus. There was no history of hypertension or cardiac disease. On arrival, the patient’s blood pressure was 165/90 mmHg, and her pulse rate was 94 beats/min. Her physical examination showed two puncture wounds on her right hand associated with ecchymosis, tenderness, and edema. Laboratory test results were within the normal range. Two vials of Zagrep antivenom (viper venom antiserum, European-equine-, Instute of Immunology, Inc., Zagreb, Croatia) was infused. Within minutes of the rate, the patient suddenly experienced full-body urticaria, facial edema, voice change, and dyspnea in which the condition was suggested as an anaphylaxis due to the antivenom theraphy. The antivenom infusion was immediately discontinued. Subcutaneous epinephrine, a dose of 0.5 mg was administered and a 1-L bolus of normal saline solution were given intravenously. Five minutes after the administration of epinephrine, a rise in blood pressure from 135/90 to 195/100 mmHg and evolution of normal heart rate (94 beats/min) to irregular tachycardia (165 beats/min) with oxygen saturation of 95% were recorded. The patient complained with dysarthria and inability to use her right hand. Electrocardiogram (ECG) showed AF with rapid ventricular response, approximately 165 beats/min. We administered intravenous amiodarone 150 mg for medical cardioversion. AF was converted to sinus rhythm after antidysrhythmic medication. A follow-up computed tomography (CT) scan of the head was normal. A bedside transthoracic echocardiogram found no structural or functional anomally. The patient’s neurologic symptoms completely resolved within three hours after hospital arrival.

## Discussion

In the present case, treatment of immediate hypersensitivity reaction via subcutaneous epinephrine was most likely caused atrial fibrillation (AF) and transient ischemic attack (TIA). The development of TIA in our case may have been due to a number of factors. It could possibly have resulted from a primary AF, directly resulting from snake venom toxicity on the myocardium. Another possibility is that the “sympathetic storm” brought on by subcutaneous epinephrine injection or snake bite-induced stress led to AF followed by TIA. In the present case, the combination of the envenomed snake bite-induced stress and epinephrine injection was considered responsible for these serious complications.

We conducted a literature review to identify documented reports of cardiac and neurologic complications due to epinephrine injections. We identified only 2 cases of cardiac (hypertensive crisis and ventricular tachycardia, atrial fibrillation) and a sole case of neurologic and cardiac complications (AF and TIA) associated with the endoscopic epinephrine injection [5-7]. Moreover, spontaneous subarachnoid hemorrhage after intravenous epinephrine use for multiple bee stings has been reported by Kwon et al. [8]. To the best of our knowledge, TIA and AF following subcutaneous epinephrine injection for the treatment of anaphlaxy in snake envenomation have not previously been noted in the literature.

In conclusion, particular care should be taken in the treatment of anaphylaxis due to snake antivenom administration, and to those with elder patients and pre-existing morbidity, such as hypertension or diabetes mellitus. The management of such condition can be complicated by cardiovascular and neurologic complications. The combination of the snake bite-induced stress and epinephrine injection which excess sympathetic stimulation may be responsible for these serious complications. 
